# The use of high-throughput small RNA sequencing reveals differentially expressed microRNAs in response to aster yellows phytoplasma-infection in *Vitis vinifera* cv. ‘Chardonnay’

**DOI:** 10.1371/journal.pone.0182629

**Published:** 2017-08-16

**Authors:** Marius C. Snyman, Marie-Chrystine Solofoharivelo, Rose Souza-Richards, Dirk Stephan, Shane Murray, Johan T. Burger

**Affiliations:** 1 The Vitis Laboratory, Department of Genetics, Stellenbosch University, Stellenbosch, South Africa; 2 Centre for Proteomic and Genomic Research, Observatory, Cape Town, South Africa; Montana State University Bozeman, UNITED STATES

## Abstract

Phytoplasmas are cell wall-less plant pathogenic bacteria responsible for major crop losses throughout the world. In grapevine they cause grapevine yellows, a detrimental disease associated with a variety of symptoms. The high economic impact of this disease has sparked considerable interest among researchers to understand molecular mechanisms related to pathogenesis. Increasing evidence exist that a class of small non-coding endogenous RNAs, known as microRNAs (miRNAs), play an important role in post-transcriptional gene regulation during plant development and responses to biotic and abiotic stresses. Thus, we aimed to dissect complex high-throughput small RNA sequencing data for the genome-wide identification of known and novel differentially expressed miRNAs, using read libraries constructed from healthy and phytoplasma-infected Chardonnay leaf material. Furthermore, we utilised computational resources to predict putative miRNA targets to explore the involvement of possible pathogen response pathways. We identified multiple known miRNA sequence variants (isomiRs), likely generated through post-transcriptional modifications. Sequences of 13 known, canonical miRNAs were shown to be differentially expressed. A total of 175 novel miRNA precursor sequences, each derived from a unique genomic location, were predicted, of which 23 were differentially expressed. A homology search revealed that some of these novel miRNAs shared high sequence similarity with conserved miRNAs from other plant species, as well as known grapevine miRNAs. The relative expression of randomly selected known and novel miRNAs was determined with real-time RT-qPCR analysis, thereby validating the trend of expression seen in the normalised small RNA sequencing read count data. Among the putative miRNA targets, we identified genes involved in plant morphology, hormone signalling, nutrient homeostasis, as well as plant stress. Our results may assist in understanding the role that miRNA pathways play during plant pathogenesis, and may be crucial in understanding disease symptom development in aster yellows phytoplasma-infected grapevines.

## Introduction

Phytoplasmas are known to infect hundreds of plant species worldwide and are responsible for devastating yield losses of many economically important crops, fruit trees, and ornamental plants [1]. They are obligate cell wall-less bacterial pathogens (class Mollicutes), and rely on plants and homopterous phloem-sucking insects for biological dispersal. In plants, they are mainly restricted to the phloem tissue where they can move and multiply through the sieve tube elements [2].

The aster yellows (AY) phytoplasma group (16SrI, subgroup A and B) represents the most diverse and widespread phytoplasma group and is also known as ‘*Candidatus* Phytoplasma asteris’ [3]. AY phytoplasma-infection can cause a severe disease in grapevine (*Vitis vinifera* L.), known as grapevine yellows (GY). Phytoplasma-like symptoms have been observed in South African vineyards since 2006, and were later shown to be caused by AY phytoplasma (16SrI-B) [4]. Transmission experiments conducted on vineyards in the vicinity of Vredendal (Western Cape) suggested that *Mgenia fuscovaria* (Hemiptera: Cicadellidae) is a vector of AY phytoplasma in South Africa [5]. GY disease incidence in the same region was monitored for different cultivars (Chenin blanc, Shiraz, Chardonnay, Cabernet Franc, Sauvignon blanc, Pinotage and Colombar), and revealed that Chardonnay is especially susceptible, based on a GY increase from 0.5% to 7.5% in two years in a single vineyard [6]. Typical symptoms caused by GY disease include discolouration and necrosis of leaf veins and laminae, downward curling of leaves, abnormal leaf shape and size, incomplete lignification, stunting and necrosis of shoots, flower abortion and berry withering. These symptoms eventually lead to reduced plant vitality and fruit yield that may hold devastating consequences for the wine and table grape industries [1,7]. Currently, the only available control strategies include early eradication of infected crops, early eradication of infected source plants (weed control), and chemical control of vectors through regular insecticide treatments [8].

*V*. *vinifera* is one of the most important fruit and/or beverage crops in the world and, like all land plants, grapevines have to develop various mechanisms at a physiological and molecular level in order to cope with their ever-changing environment. Significant progress has been made to understand plant-pathogen interactions and the multiple gene regulatory mechanisms they invoke during plant defence responses. The recent successful, axenic cultivation of phytoplasmas [9] will allow direct *in planta* investigation of molecular interactions postulated to exist between phytoplasmas and their plant and insect vectors. In addition, high-throughput transcriptome analysis of next-generation sequencing (NGS) and microarray data, as well as proteomics, have served as valuable approaches for gaining new insights into physiological, biochemical and molecular mechanisms underlying phytoplasma disease symptom development in grapevine and other plant species [10–16].

Increasing evidence has shown that a class of small non-coding endogenous RNAs known as microRNAs (miRNAs), play a major role in post-transcriptional gene regulation during plant development and plant responses to biotic and abiotic stresses [17,18]. Mature miRNAs are typically 19 to 24 nt in length and originate from miRNA (*MIR*) genes that are transcribed by RNA Polymerase II. These transcripts, known as primary miRNAs (pri-miRNA), form imperfect fold-back hairpins that are cleaved by RNase III-like Dicer 1 (DCL1) to produce miRNA precursors (pre-miRNA). Each pre-miRNA contains one or more short intermediate complementary miRNA/miRNA* duplexes. These duplexes are then cleaved by DCL1 from the stem region and processed inside the nucleus to be exported to the cytoplasm where the leading miRNA is incorporated into the RNA-induced silencing complex (RISC). When associated with the RISC, guided binding of the miRNA to its complementary target mRNA(s) or non-coding trans-acting siRNA (*TAS*) transcript(s) occurs. This facilitates either translational inhibition or degradation of target mRNA(s), or slicing of *TAS* transcripts that lead to generation of trans-acting siRNAs (tasiRNAs). Target degradation occurs through endonucleolytic cleavage by the RISC core protein ARGONAUTE 1 (AGO1) [19–21].

It has been suggested that the miRNA pathway contributes to pathogen-associated molecular pattern (PAMP)-triggered immunity (PTI), which refers to a basal defence response upon recognition of certain pathogenic elements, such as flagellin [22]. The bacterial PAMP peptide flg22 causes induced expression of the *Arabidopsis* miR393, which was the first miRNA identified to play a role in plant PTI. Overexpression of miR393 caused down-regulation of auxin receptor mRNAs, including *transport inhibitor response 1* (*TIR1*), through degradation, which caused increased resistance to virulent *Pseudomonas syringae* pv. *tomato* (*Pst*) DC3000 [23].

The availability of two draft *V*. *vinifera* cv. ‘Pinot noir’ genome sequences obtained from NGS projects [24,25] has enabled rapid discovery of miRNAs that further supports efforts to explore small RNA (sRNA)-based regulatory networks in grapevine. The use of computational analyses of high-throughput sequencing and microarray data, followed by experimental validation, have been used to identify highly conserved miRNAs, some of which play important roles in grapevine development [26,27]. To date, 186 mature grapevine miRNA sequences from 47 different miRNA families have been deposited in miRBase v21 [28].

This study is the first to utilise a bioinformatics pipeline to dissect complex high-throughput sRNA sequencing (sRNA-seq) data in order to identify miRNAs that are differentially expressed in *V*. *vinifera* cv. ‘Chardonnay’ in response to AY phytoplasma-infection. Furthermore, we used computational resources for the *in silico* prediction and annotation of putative miRNA targets to explore the involvement of possible pathogen response pathways. Understanding sRNA-mediated gene regulation is crucial to expanding our knowledge of gene regulatory pathways involved in different stress-regulated physiological processes. Our results provide insight into miRNA-mediated pathogenesis in *V*. *vinifera* and may shed light on disease control strategies for molecular breeding in the future.

## Materials and methods

### Plant material

**Fig 1:**
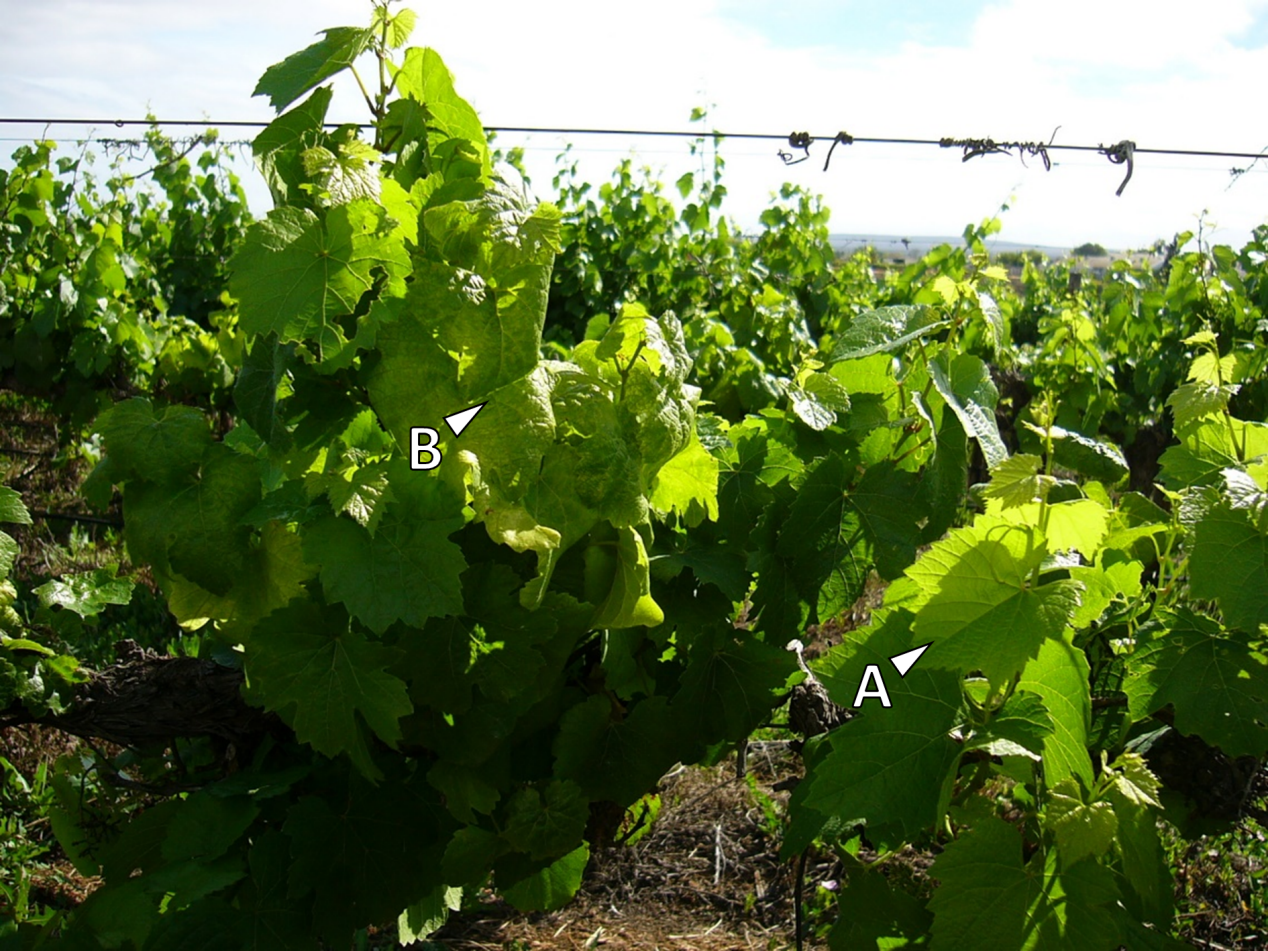
*Vitis vinifera* cv. ‘Chardonnay’ with asymptomatic leaves (A), and leaves showing typical aster yellows (AY) disease symptoms (B).

We visually selected and tagged 50 symptomatic and 50 asymptomatic *V*. *vinifera* cv. ‘Chardonnay’ plants in a 7-year-old vineyard in the Olifants River Valley (Western Cape) (Fig 1). The vineyard was part of a high disease incidence area mapped by the Agricultural Product Inspection Services (APIS) of the Department of Agriculture, Forestry and Fisheries (DAFF). Permission was granted by the owner to conduct the study on his farm, Daltana. During the peak summer season, whole leaf material, including the blade and petiole, were collected from each plant, immediately flash frozen in liquid nitrogen, transported on dry ice and stored at -80°C until use. RNA was extracted using a modified CTAB method [29], while genomic DNA was extracted using a NucleoSpin^®^ Plant II kit (Macherey-Nagel; Düren, Germany). Phytoplasma infection was confirmed by a nested-PCR procedure, specifically amplifying a region of the phytoplasma 16S rDNA. The first PCR round was performed using a universal primer pair R16mF2/mR1, followed by a second PCR with the R16F2n/R2 primer pair [30]. Afterwards samples were screened for the most prevalent grapevine viruses, including Grapevine leafroll-associated virus 3 (GLRaV-3), Grapevine virus A (GVA), Grapevine virus E (GVE), and Grapevine rupestris stempitting-associated virus (GRSPaV), using two-step RT-PCR assays. Primer sequences for virus screening were obtained from previous publications (S1 File). Results from these diagnostics were used to select material, free from these viruses, from three AY phytoplasma-infected, and three healthy plants for further experiments.

### Total RNA extraction and sRNA-seq

Large-scale RNA extractions were carried out on one gram of plant material for each of the six experimental plants using PureLink^®^ Plant RNA Reagent (Thermo Fisher Scientific, Waltham, Massachusetts, United States), according to the manufacturer’s protocol, with an additional phenol-chloroform extraction step when further purification was necessary. Total RNA was quantified on a NanoDrop ND-1000, while RNA integrity was assessed using a Plant RNA Nano Assay using an Agilent 2100 Bioanalyzer. Ten micrograms of total RNA from each plant were sent to Fasteris SA (Plan-les-Ouates, Switzerland) for sRNA-seq. The six sRNA libraries were constructed using the TruSeq® Small RNA Library Prep Kit protocol (Illumina, San Diego, California, USA), followed by sRNA-seq on an Illumina HiSeq2000 platform (https://support.illumina.com).

### sRNA bioinformatic analysis

After sRNA-seq, high-quality, adapter-trimmed sequence data was received from the service provider in Illumina-fastq format. FastQC (www.bioinformatics.babraham.ac.uk/projects/fastqc/) was used as a tool to visualise different quality control measurements. In order to confirm RT-PCR results of the virus screening, we produced *de novo* assemblies with the 18 to 26 nt sRNA reads of each sample, using Velvet v1.1 [31]. The resulting contigs were compared against the NCBI database using nucleotide BLAST [32].

The unique (non-redundant) 18 to 26 nt sequences with accompanying copy numbers, across all six libraries (representing the six biological samples), were submitted to miRanalyzer [33] (http://bioinfo5.ugr.es/miRanalyzer/miRanalyzer.php) for known miRNA analysis, allowing one mismatch. All reads that mapped to other non-coding RNAs (ncRNAs) in RFam (http://www.sanger.ac.uk/science/tools/rfam) and RepBase (http://www.girinst.org/repbase/) were removed, and the remaining reads were mapped against the canonical grapevine miRNA (vvi-miRNA) sequences deposited in miRBase v21. Mapped read counts for libraries obtained from the phytoplasma-infected group were compared to those from the healthy (control) group using the DESeq v2 package for differential expression analysis [34] (http://bioconductor.org/packages/release/bioc/html/DESeq.html).

For novel miRNA predictions, sRNA library files of the 18 to 26 nt reads, from all six libraries, were grouped into a single file that served as input for sRNAbench v0.9 [35] (http://bioinfo5.ugr.es/srnatoolbox/srnabench), and Shortstack v0.4.1 [36], using the default parameters of the respective packages. sRNAbench was also used for the discovery of sequence variants of known miRNAs, also known as miRNA isoforms (isomiRs). The *V*. *vinifera* (cv. ‘Pinot noir’; PN40024) 12x coverage genome assembly (http://www.genoscope.cns.fr/externe/GenomeBrowser/Vitis/) served as the reference sequence to which the sRNA reads were mapped [24]. Importantly, primary criteria described by Meyers *et al*. [37] for duplex-forming precursors (pre-miRNAs) are used by both programs. These include that (1) the miRNA and miRNA* are derived from opposite arms within the stem region to form a duplex with two 3’-nucleotide overhangs; (2) extensive base-pairing exist between the miRNA and the other arm of the hairpin, which includes the miRNA*; and (3) asymmetric bulges are minimal in size and frequency, especially within the miRNA/miRNA* duplex.

The Unified Nucleic Acid Folding (UNAFold) software was used to calculate the minimum folding free energy (MFE; Δ*G*) of novel pre-miRNA sequences [38] (http://mfold.rna.albany.edu/). In an effort to find more comprehensive evidence that miRNAs differ from other RNAs, Zhang *et al*. [39] described a statistical method incorporating pre-miRNA folding free energies, base pairing, nucleotide composition, and other characteristics. This method was defined by two criteria known as the adjusted minimum folding free energy (AMFE) and the minimal folding free energy index (MFEI). The AMFE and MFEI were calculated using the following equations:

$$AMFE=\frac{MFE}{Precursor\;length\;(nt)}\times 100$$

$$MFEI=\frac{AMFE}{\%GC\;content\;of\;precursor}$$

Precursor sequences were analysed in RNAfold to view their stem-loop secondary structures [40]. Novel mature miRNA sequences were compared against the miRBase v21 database using BLASTn v2.2.29+ [32,41] (http://www.ncbi.nlm.nih.gov/books/NBK1763/) for the identification of miRNA homologs. Only the top BLAST results, with an identity of ≥90%, zero gaps and not more than two mismatches (over a seed region of 18 nt), were regarded as homologs. For each resulting BLAST hit, we compared the associated precursor sequence against miRBase with the miRBase BLASTn tool, using less stringent parameters, to identify homologous pre-miRNA sequences.

The number of sRNA reads that aligned to novel mature miRNA sequences present in all six libraries were obtained with Bowtie v1.0.1 [42] and a customised shell script. The resulting count data were analysed in DESeq v2 to obtain differentially expressed novel miRNAs. Only log_2_-fold changes with an adjusted *p*-value of ≤ 0.05 were considered significant.

### Validation of miRNA expression by real-time RT-qPCR

Stem-loop reverse transcription quantitative PCR (RT-qPCR) assays were performed according to the methods of Chen *et al*. [43] to validate the DESeq differential expression results. High-quality total RNA was prepared as described above. For each miRNA a 20 μl reverse transcription reaction was prepared containing 100 U of Superscript III reverse transcriptase (Invitrogen, Carlsbad, CA, USA), 20 U of RiboLock RNase inhibitor (Thermo Scientific, Waltham, Massachusetts, United States), 4 μl first-strand buffer (5x), 5 mM DTT, 500 nM dNTPs and 1 μl miRNA-specific stem-loop RT primer (10 μM) and 1.2 μg total RNA. Cycling conditions were as follows: 30 min at 16°C, 60 cycles at 30°C for 30 s, 42°C for 30 s, and 50°C for 1 s, heat inactivation for 5 min at 85°C, and cooling at 4°C. qPCR was performed using the Universal ProbeLibrary (UPL) probe assay with UPL probe #21 (Roche Diagnostics, Basel, Switzerland). Each 10 μl reaction mixture was prepared in triplicate and contained 1 μl cDNA, 5 μl FastStart TaqMan^®^ Probe Master (2x) (Roche Diagnostics, Basel, Switzerland), 0.5 μl miRNA-specific forward primer (10 μM), 0.5 μl universal reverse primer (10 μM), 0.1 μl UPL probe (10 μM), and nuclease-free water. A control reaction, without cDNA template, was included for each miRNA. Based on previous results from geNorm analysis (qBase^PLUS^ v2.0, Biogazelle, Ghent, Belgium) [44], miR167a was chosen as internal control to normalise miRNA expression levels (data not shown). PCR amplification was performed in an Applied Biosystems 7900HT Fast Real-Time PCR System, in which the baseline and threshold cycles (C_t_) were automatically determined with SDS v2.3 software. Cycling conditions were as follows: 95°C for 5 min, 45 cycles at 95°C for 10 s and 60°C for 1 min. Relative miRNA expression analysis was performed using qBase^PLUS^ v2.0 software (Biogazelle, Ghent, Belgium).

### miRNA target prediction and functional annotation

Potential targets of differentially expressed miRNAs were predicted using the psRNAtarget analysis server [45] (http://plantgrn.noble.org/psRNATarget/), with default parameters which included a threshold cut-off of 3.0 for low false-positive prediction, a complementarity scoring length of 20 bp, and the energy required for target accessibility equal to 25 kcal/mole. The collection of annotated transcript sequences of the *V*. *vinifera* (PN40024) 12x assembly was used for the miRNA target search (http://www.genoscope.cns.fr/externe/GenomeBrowser/Vitis/). Predicted targets for both the known and novel differentially expressed miRNAs were functionally annotated using Blast2GO v2.2.7 [46]. This was done by using NCBI BLASTx to find homologous sequences, a mapping step to retrieve gene ontology (GO) terms associated with BLAST hits (http://geneontology.org/page/go-database), and assigning functional attributes to each query sequence in terms of biological processes, cellular components and molecular functions, in a species-independent manner. Afterwards a combined graph was generated using a GO sequence similarity level of 3 and an annotation cut-off value of 7.

## Results and discussion

### Plant material

According to the diagnostic PCR screening results (data not shown), 19 out of the 50 plants that were visually tagged as ‘healthy’ were AY phytoplasma-positive, while 32 out of the 50 plants that were visually tagged as phytoplasma-infected were confirmed positive for AY phytoplasma. The remaining 31 ‘healthy’ (no phytoplasma-infection) and 32 phytoplasma-infected candidate plants were subjected to further virus screening. All plants that tested positive, following the virus-screening, were eliminated from the study. BLAST results for the *de novo* assembled contigs also confirmed the absence of any prevalent grapevine viruses (data not shown). Our final test groups consisted of three phytoplasma and virus-free Chardonnay plants for the control group (h55, h85, h89), and three AY phytoplasma-infected, but virus-free, Chardonnay plants for our experimental group (p73, p93, p99).

### sRNA-seq

To investigate miRNA expression profiles in response to GY disease, individual sRNA libraries were constructed from RNA extracted from pooled leaf material of the six plants. High-quality, adapter-trimmed reads were generated from the respective sRNA-seq libraries and the number of reads are displayed in Table 1.

**Table 1:** Summary of total small RNA reads.

	Small RNA library type	
Total high-quality reads	Healthy	AY
p73	N/A	10,893,265
p93	N/A	10,476,093
p99	N/A	10,511,436
h55	10,878,402	N/A
h85	12,424,487	N/A
h89	11,510,533	N/A
All	34,813,422	31,880,794
18–26 nt	26,474,279	24,314,330
18–26 nt: unique	6,388,422	5,726,632
18–26 nt: mapped	22,515,584	20,782,176

H: Health (control) sample group

AY: AY phytoplasma-infected sample group

N/A: Not applicable

**Fig 2:**
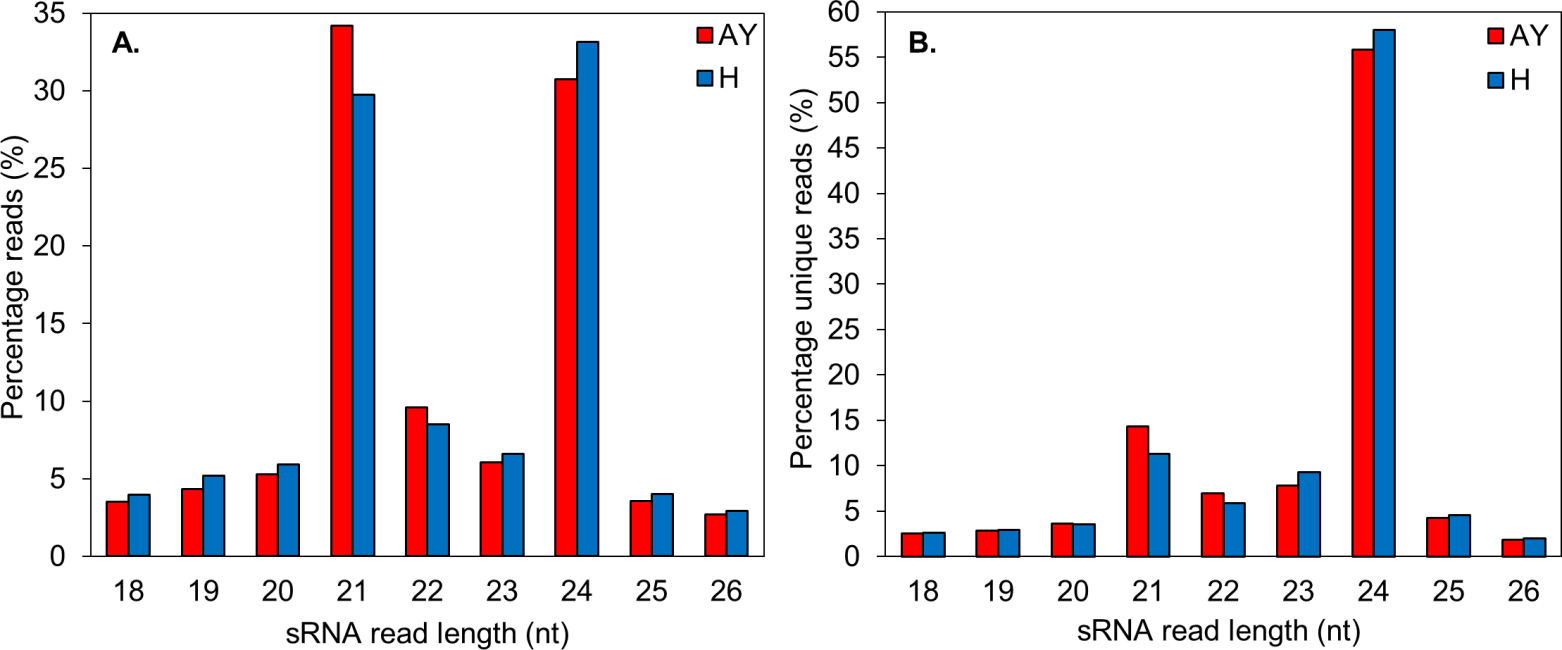
(A) The size distribution of the total 18 to 26 nt sRNA reads in the healthy (H) and AY phytoplasma-infected (AY) libraries. (B) The size distribution of the total 18 to 26 nt unique sRNA reads in the healthy (H) and AY phytoplasma-infected (AY) libraries.

Analysis of the size distribution of sRNA sequences in the 18 to 26 nt range showed the most abundant sequences to be between 21 and 24 nt in length, with sizes 21 nt and 24 nt as the major classes (Fig 2). These results were consistent with those of other grapevine cultivars, as well as *Arabidopsis*, *Citrus trifoliate*, *Oryza sativa*, *Eugenia uniflora*, and *Glycine max* [26, 47–51]. The library generated from the phytoplasma-infected samples indicated that 21 nt sRNAs were more abundant (34.2%) than those in the library obtained from the healthy plant samples (29.7%). A similar profile was observed for Mexican lime infected with ‘*Candidatus* Phytoplasma aurantifolia’ [52]. The 24 nt sRNAs, however, were more abundant in the library from the healthy plant samples (33.2%) compared to the library from the phytoplasma-infected samples (30.7%). This observation points to differences in complexity between the two pools of sRNAs that may infer an underlying miRNA-mediated regulatory response triggered by biotic stress. The unique (non-redundant) 21 nt reads were also more abundant in the phytoplasma-infected samples. Their length is characteristic of canonical miRNAs, and they possessed a high reads/unique reads ratio (Fig 2), reflecting their regulatory impact and abundance in plants. The 24 nt reads, which are predominantly repeat-associated siRNAs (rasiRNAs), exhibited the highest sequence diversity, consistent with the origin of this size class (Fig 2B) [53].

### Identification of known miRNAs and their sequence variants

We used sRNAbench v0.9 to detect both the canonical vvi-miRNA sequences (from miRBase), and all isomiRs present in the pooled phytoplasma-infected and healthy (control) read data, respectively. The different sequences were classified and are presented in a simple table output (S2 File). IsomiRs are defined as different sequence variants of known miRNAs that may arise from post-transcriptional modifications and alternative processing [54]. IsomiR types included those reads having non-template additional nucleotides (where the read sequence starts and ends at the same position as the canonical sequence in the pre-microRNA, but shows sequence variation), “flush fitting” length variants (where the read sequence always starts or ends at the same position as the canonical sequence but a terminal trimming or extension is evident), and multiple length variants (where the read sequence does not coincide with either the 3’ or 5’ terminal nucleotides of the canonical sequence). Those reads that contained the same 5’ terminal nucleotides as the canonical vvi-miR166b sequence, but showed divergence of length in their 3’ terminal extension, as a result of alternative DCL1 cleavage, were the dominantly expressed isomiRs in the ‘healthy libraries’ (S2 File). In the case of the ‘AY phytoplasma-infected libraries’, those reads that contained the same 3’ terminal nucleotides as the canonical vvi-miR166e-5p sequence, but showed divergence of length in their 5’ terminal extension, as a result of alternative DCL1 cleavage, were the most dominantly expressed isomiRs (S2 File).The mechanism by which miRNA heterogeneity arises has been extensively reviewed. Different findings have suggested that multiple isomiRs that arose from a single miRNA locus are not randomly generated artefacts, but rather generated *in vivo* through biological relevant processes. Consequently, such sequence variations may drastically alter miRNA association with their targets, and also influence miRNA stability during Argonaut (AGO)-RISC loading [55–57].

The vvi-miR166 family showed the highest levels of expression, but had no significant difference in terms of the total normalised read counts between the two different library types (S2 File). Vvi-miR166b and its isomiRs constituted ~40% of the total normalised read counts in both library types. This high level of vvi-miR166 expression was also seen in a previous study where it was the most dominantly expressed miRNA family in all assayed grapevine tissues [26]. A degradome sequencing approach revealed that vvi-miR166b regulates a Class III homeodomain leucine zipper (HDZIP-III) transcription factor which is involved in secondary cell wall biosynthesis [25,58,59]. Direct evidence from the identification and analysis of corresponding activation tagged mutants has implicated the regulatory involvement of miR165/166 in leaf and vascular morphogenesis [60,61].

### Differential expression analysis of known miRNAs

**Fig 3:**
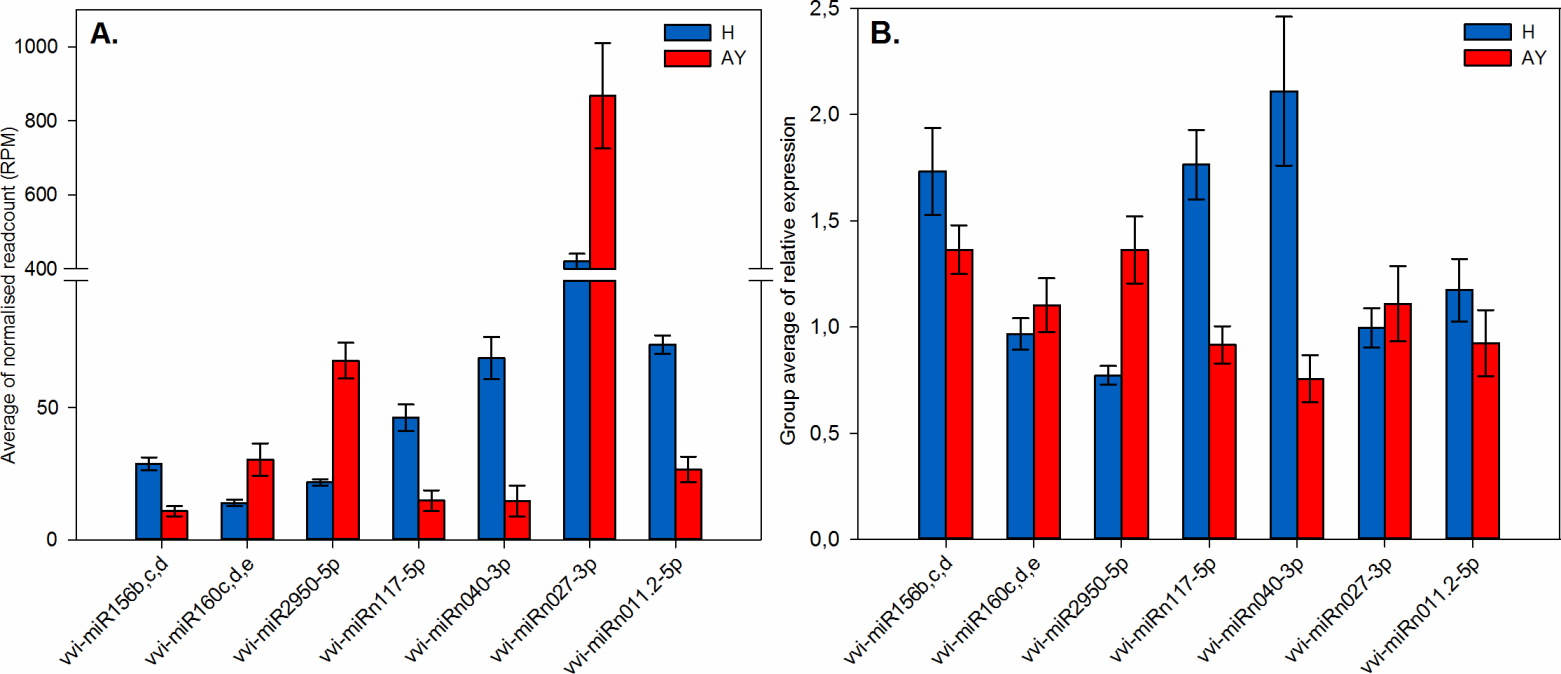
Bar charts displaying profiles of differentially expressed vvi-miRNAs (*q* ≤ 0.05) in healthy (H) and AY phytoplasma-infected (AY) samples that were further validated. **Vertical bars indicate the standard error (SE) of the mean.** (A) Average normalised read counts of vvi-miRNAs. Group averages were given in terms of the average of reads per million mapped reads (RPM) for three biological replicates. (B) Relative expression analysis with real-time RT-qPCR, confirming expression profiles of vvi-miRNAs. Each bar represents the average of three biological replicates with three technical replicates.

Comparative profiling, with DESeq v2, between the healthy (control) and AY phytoplasma-infected samples was used to determine the differential expression of known miRNAs in the AY phytoplasma-infected material. Based on false discovery rate (FDR) for multiple testing, we encountered seven significantly differentially expressed known vvi-miRNA families that had log_2_-fold changes with adjusted *p*-values (*q*) ≤ 0.05. (Fig 3, Table 2).

**Table 2:** List of significantly differentially expressed known vvi-miRNAs.

Kown miRNA	Sequence (5'-3')	Length (nt)	Avg of normalised read counts^†^		DESeq results (H vs AY)		
			H	AY	log_2_FC	*p*-value	Adj. *p*-value (*q*-value)
vvi-miR156b,c,d^¶^	UGACAGAAGAGAGUGAGCAC	20	29.29	11.06	-1.18	0.0011	0.0102
vvi-miR159c	UUUGGAUUGAAGGGAGCUCUA	21	3869.83	8860.93	1.15	3.89E-05	0.0007
vvi-miR399g	UGCCAAAGGAGAUUUGCCCCU	21	463.24	103.94	-2.02	3.89E-05	0.0007
vvi-miR171a,c,d,I,j	UGAUUGAGCCGUGCCAAUAUC	21	20.56	37.71	0.87	0.0006	0.0071
vvi-miR172d	UGAGAAUCUUGAUGAUGCUGCAU	23	243.48	736.45	1.42	0.0007	0.0075
vvi-miR160c,d,e^¶^	UGCCUGGCUCCCUGUAUGCCA	21	14.23	30.9	1.00	0.0060	0.0477
vvi-miR2950-5p^¶^	UUCCAUCUCUUGCACACUGGA	21	22.15	69.15	1.59	3.26E-10	2.35E-08

H: Healthy sample group

AY: AY phytoplasma-infected sample group

^¶^Validated using real-time RT-qPCR

^†^Average of reads per million mapped reads (RPM) between three biological replicates

An additional nine known miRNAs from seven families, had log_2_-fold changes with significant *p*-values (*p* ≤ 0.05), which indicate they may be of biological importance (S3 File). A total of eight miRNA families, *viz*. vvi-miR159c, vvi-miR160c-e, vvi-miR171acdij, vvi-miR172d, vvi-miR2950-5p, vvi-miR319bcef, vvi-miR3627-5p, and vvi-miR395a-m, were up-regulated, and five, *viz*. vvi-miR156bcd, vvi-miR3629(a-3p, b-3p, c-5p), vvi-miR3638-5p, vvi-miR399aheg, vvi-miR479, were down-regulated (Table 2, S3 File). The differential expression of conserved miRNA families (vvi-miR156, miR159, vvi-miR160, vvi-miR171, vvi-miR172, vvi-miR319), known to be involved in different aspects of plant development [18], make these potential candidates that play a role in the interactions leading to symptoms associated with GY.

### Novel miRNA prediction and differential expression analysis

The pooled sRNA reads from all six libraries served as input for sRNAbench v0.9 and Shortstack v0.4.1 for predicting novel miRNAs. These sRNA sequences were aligned to the *V*. *vinifera* (PN40024) 12x assembled genome sequence to identify loci that may harbour potential pre-miRNA sequences, based on secondary structure and read distribution. Known *V*. *vinifera* pre-miRNA chromosomal locations found in miRBase v21 were flagged during each analysis to obtain unique precursor sequences that did not match these loci. Secondary fold structures were viewed using RNAfold and all miRNA precursors displayed appropriate stem-loop hairpin secondary structures (data not shown). Based on structural criteria described by Meyers *et al*. [37], these miRNAs can be regarded as authentic candidates that adhere to biogenesis and expression criteria for confident miRNA annotation.

In total, 175 novel pre-miRNA sequences were predicted, each derived from a unique genome location (S4 File). Three of the pre-miRNAs were predicted with both prediction pipelines. We also identified multiple pre-miRNAs that produce mature miRNAs with similar sequences, e.g. vvi-miRn024a to vvi-miRn024c.These miRNAs can be considered members of the same miRNA family (S4 File). Likewise, vvi-miRn019a to vvi-miRn019g represent a larger family of duplicated miRNA paralogs with identical precursor and mature miRNA sequences (S4 File).

**Fig 4:**
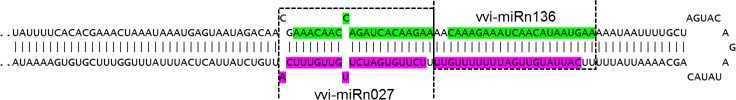
An example of a novel pre-miRNA hairpin structure that may give rise to two different miRNA duplexes (See S4 File). The sequences highlighted in green and magenta represents the 5’ and 3’ mature miRNA sequences, respectively.

Pre-vvi-miRn027, predicted with Shortstack, may serve as an example of a large precursor that could give rise to two different miRNA duplexes since the sRNAbench-predicted pre-vvi-miRn136 falls within its location (Fig 4; S4 File). Precursor sequences ranged from 54 nt to 742 nt while mature miRNA sequences ranged from 20 nt to 25 nt in length, the majority being 21 nt. Most mature miRNA sequences started with an uracil at the first position, corroborating data described by Baumberger and Baulcombe [62] that showed a preferential association of the AGO1 protein with sRNAs containing a 5’-terminal uracil. This may indicate an important characteristic for miRNA biogenesis through recognition of miRNA duplexes by RISC.

Zhang *et al*. [39], implemented a criterion to better distinguish miRNAs from other sRNAs, known as MFEI which incorporates MFE, sequence length and GC content. The Unified Nucleic Acid Folding (UNAFold) software was used to calculate the MFE. MFE for predicted novel pre-miRNAs ranged from -13.2 kcal/mol to -428.9 kcal/mol and the MFEI ranged from -0.47 to -2.51. The majority (>94%) of the novel Shortstack-predicted pre-miRNAs possessed MFEI-values in accordance with the expected value (≥-0.85), while only 39% of sRNAbench-predicted pre-miRNAs had strong negative MFEI-values of more than -0.85 (S4 File).

Each novel pre-miRNA sequence, as well as its most abundant mature miRNA sequence, was subjected to a homology-based search against miRBase using BLASTn. Results indicated that some of the newly identified pre-miRNAs have either precursor and/or mature sequences homologous to conserved miRNAs from other plant species, including grapevine miRNAs. Vvi-miRn025a and vvi-miRn025b, for example, share high homology with vvi-miR171 (S4 File). We identified 17 novel vvi-miRNAs belonging to 13 miRNA families that can be classified as newly-identified members of known miRNAs, based on homology to known plant miRNAs for both precursor and mature sequences (S4 File).

Differential expression analysis revealed that 10 novel miRNAs were significantly up-regulated, while 13 novel miRNAs were significantly down-regulated in sRNA libraries prepared from AY phytoplasma-infected leafmaterial (Table 3). We used a Perl script provided by Shen *et al*. [63] to generate hairpin structures of these differentially expressed novel miRNAs (S1 Fig). These images demonstrated complementary 5’ and 3’ mature miRNA sequences, each within a duplex that was possibly DCL1-derived.

**Table 3:** List of significantly differentially expressed novel vvi-miRNAs.

Novel miRNA	Sequence (5'-3')	Length (nt)	Avg of normalised read counts^†^		DESeq results (H vs AY)		
			H	AY	log_2_FC	*p*-value	Adj. *p*-value (*q*-value)
vvi-miRn010.2-3p (miR529_new)	GCUGUACCCUCUCUCUUCCCC	21	8.71	2.20	-1.58	3.88E-07	5.39E-05
vvi-miRn025b/n025a-3p (miR171_new)	UGAUUGAGCCGUGCCAAUAUC	21	20.56	37.71	0.93	4.40E-07	5.39E-05
vvi-miRn011.2-5p^¶^ (miR391_new)	AGGAGAGAUGACGCCGUCGCC	21	75.28	27.16	-1.23	9.60E-07	7.84E-05
vvi-miRn133-5p	AGACUGGUAGAAAGAUUUAUA	21	19.36	2.73	-1.78	7.27E-06	4.45E-04
vvi-miRn140-3p	UCACCUUGUUGAGUGCCCGGU	21	6.48	1.59	-1.50	1.29E-05	6.31E-04
vvi-miRn040-3p^¶^	UGGGUUCAAAGUAGACAAUAUUUA	24	70.10	14.94	-1.57	2.09E-05	8.53E-04
vvi-miRn131-3p (miR399_new)	UGCCAAAGGAGAUUUGCCCCG	21	2.73	0.53	-1.56	4.22E-05	1.48E-03
vvi-miRn117-5p^¶^	UGGACCCUCAUGACUUUAAAAUGC	24	47.07	15.09	-1.29	6.07E-05	1.86E-03
vvi-miRn139-3p	GGGGGCUGACCUGUUGAAGAG	21	21.50	8.60	-1.04	0.0002	0.0045
vvi-miRn150-5p	UUUUUCAUGGUCUGAUUGAGC	21	15.97	36.25	1.11	0.0002	0.0045
vvi-miRn022b-5p (miR1446_new)	UCUGAACUCUCUCCCUCAUUGGC	23	0.76	2.45	1.35	0.0002	0.0045
vvi-miRn008.1-3p (miR169_new)	AGGCAGUCACCUUGGCUAACU	21	3.72	1.17	-1.22	0.0004	0.0081
vvi-miRn147-5p	UGGUGAACCAAAUAACUCUGG	21	33.29	63.81	0.93	0.0009	0.0174
vvi-miRn027-3p^¶^	UCUUGUGAUCUUGUUGUUUCA	21	420.78	867.56	0.99	0.0010	0.0174
vvi-miRn115-3p	AGGAAUGUGCUUCUUGGCAUA	21	6.45	1.84	-1.19	0.0016	0.0261
vvi-miRn070-3p	UAAGGACUAAAUUGGUAGACC	21	1.92	4.03	0.97	0.0022	0.0334
vvi-miRn089-5p	UACACAUGUAGUGCCAUCAUAUGA	24	53.03	16.67	-1.13	0.0025	0.0365
vvi-miRn007.1-3p	UGAUAUUAGCAGCUGAGAACA	21	7.19	3.71	-0.76	0.0032	0.0386
vvi-miRn003-5p	UUACACAGAGAGAUGACGGUGG	22	24.78	53.83	0.98	0.0031	0.0386
vvi-miRn051-5p	AGAGACCACCUAGUCCUGUUAAGA	24	31.20	19.81	-0.52	0.0029	0.0386
vvi-miRn129-5p	UUUUGGAACUAGAGUGCUUGC	21	1.34	2.89	0.98	0.0035	0.0410
vvi-miRn137 -5p	CAACAAUCUAAAUGAAACAUAGA	23	3.40	6.61	0.90	0.0043	0.0478
vvi-miRn022a-5p (miR1446_new)	UCUGAACUCUCUCCCUCAUGGC	22	8.36	15.35	0.85	0.0047	0.0497

H: Healthy sample group

AY: AY phytoplasma-infected sample group

^¶^Validated using real-time RT-qPCR

^†^Average of reads per million mapped reads (RPM) between three biological replicates

Known miRNA homologs are given in brackets

### Validation of miRNA expression profiles by real-time RT-qPCR

**Fig 5:**
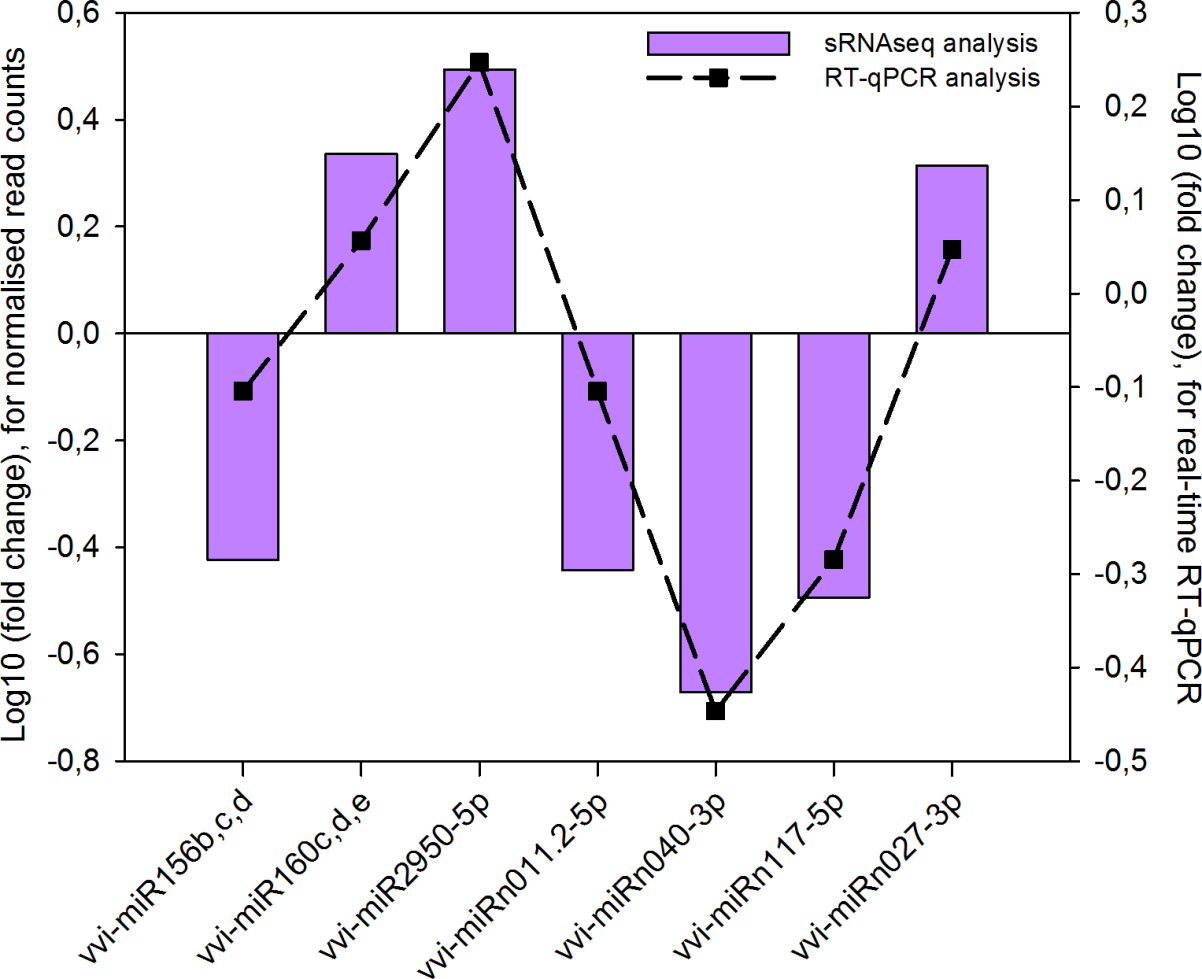
Correlation graph comparing average normalised read counts with real-time RT-qPCR results, thereby confirming vvi-miRNA expression patterns.

A stem-loop RT-qPCR assay was applied to verify the results for the miRNA differential expression analysis. Primers sequences are listed in the S5 File. The relative expression of seven significantly differentially expressed miRNAs, three known (*viz*. miR156bcd, miR160cde, and miR2950-5p) and four novel (*viz*. miRn011.2-5p, miRn040-3p, miRn117-5p, and miRn027-3p), was measured in healthy and phytoplasma-infected leaves using real-time RT-qPCR analysis (Fig 3B). We were also able to validate the expression of less significant known miRNAs (*q* ≤ 0.15) (*viz*. miR319e, miR399e, and miR479) (S3 File). This suggests that modulation of these miRNAs may hold biological importance. Although the non-conserved miRNAs (*viz*. vvi-miR479 and vvi-miR2950), and certain novel miRNAs were present at low levels, they were detected using real-time RT-qPCR. The trend of expression obtained from the RT-qPCR analysis was consistent with the average normalised read abundance observed in the sRNA-seq data (Fig 5; S3 File). Since the expression of the novel miRNA candidates were confirmed using real-time RT-qPCR they can be tentatively classified as authentic miRNAs. The use of stable and robust degradome data, however, will provide us with more concrete evidence to confirm these results [64].

### Identification of putative targets for differentially expressed known miRNAs

Over the past decade, increasing evidence have demonstrated how miRNAs can play an important role in modulating gene expression during plant-microorganism interactions [65]. It is important to consider miRNA target identification and validation in order to elucidate the biological functions of miRNAs. Multiple ‘Pinot noir’ target mRNAs have been identified for known miRNAs using a high-throughput degradome sequencing approach [26]. To gain further insight into the function of the differentially expressed miRNAs found in this study, we performed a complementary-based search with psRNAtarget to search for putative target-binding sites found in grapevine mRNAs. We adopted strict parameters, which provided perfect or near-perfect complementarity between a miRNA and its target, suggesting DCL1-cleavage or translational inhibition of miRNA-targeted mRNAs [66,67] (S6 File).

**Fig 6:**
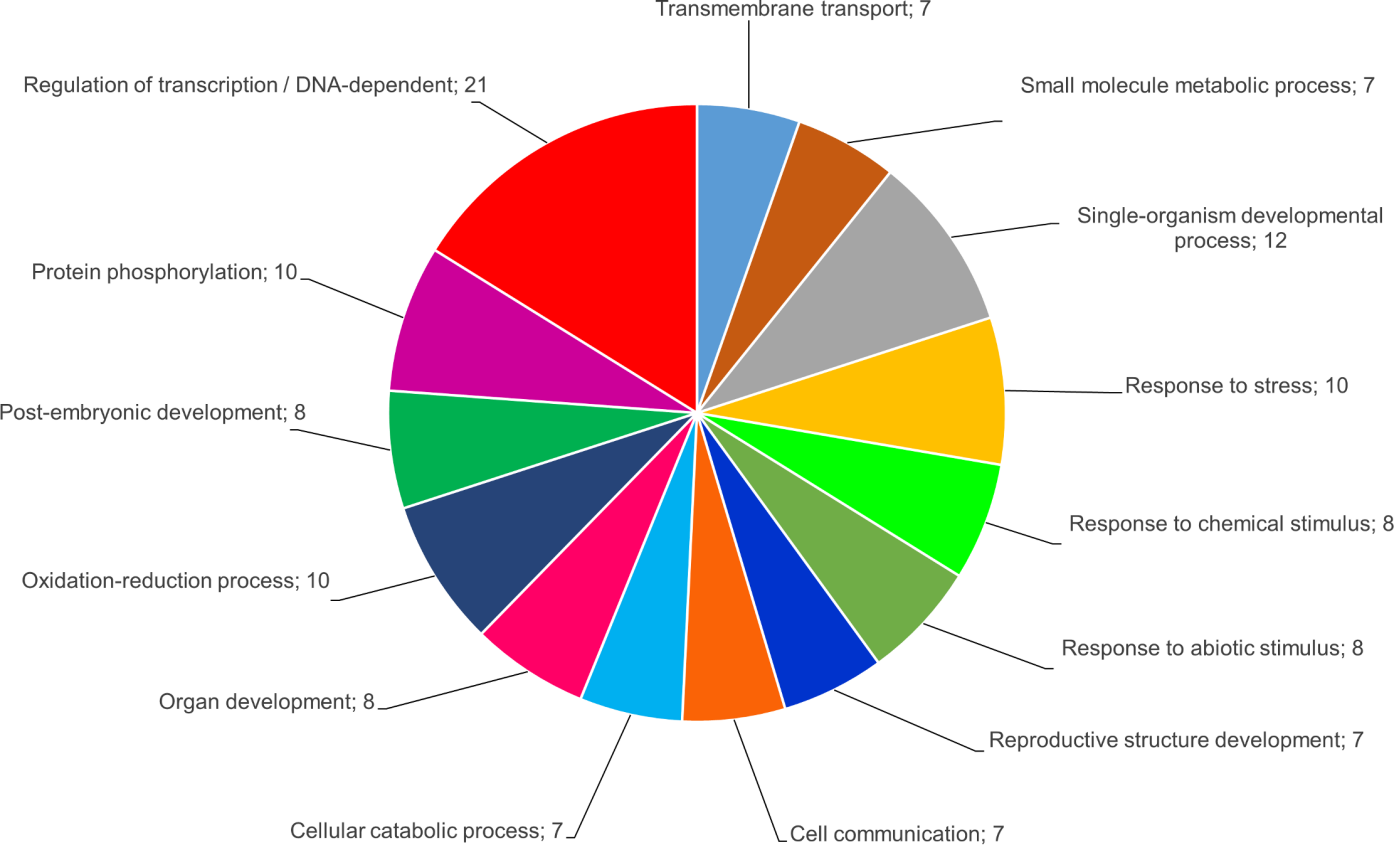
A combined graph depicting the main categories of putative vvi-miRNA targets grouped in terms of biological processes (GO level 3; annotation cut-off = 7.0).

In order to obtain a holistic view of biological pathways possibly influenced by miRNA-mediated regulation, *in silico* predicted targets for both the differentially expressed known and novel miRNAs were functionally annotated using Blast2GO v2.2.7. After GO analysis we found 71 functionally annotated putative targets for 15 of the known miRNAs and 54 functionally annotated putative targets for 17 of the novel miRNAs. For some of the targets, however, functional attributes could not be assigned, using default parameters within Blast2GO. Detailed annotation results are provided in the S6 File. A combined graph was generated and depicted different categories in which the targets grouped in terms of biological processes (Fig 6).

There were 14 categories of which the four major processes included transcriptional regulation, developmental processes, response to stress, and metabolic processes that included phosphoregulation and oxidoreductase activity. This suggested that the differentially expressed miRNAs are involved in a broad range of physiological functions. Putative targets of conserved miRNA families, such as miR156, miR159, miR171 and miR399, identified in this study, correspond to targets found in numerous plant species, including several grapevine cultivars, while the predicted functions of these targets were also similar with previous findings [26,27,58,60,68–70] (S6 File). Recent studies have revealed that several of these miRNA targets share common roles in the crosstalk between signalling pathways modulated by both biotic and abiotic stresses [71]. Our results revealed that some of these target genes encode transcription factors, including squamosa-promoter binding protein (SPB)-box, MYB, NAC-domain, Scarecrow-like/GRAS-domain, AP2, HDZIP-III and bHLH transcription factors, previously reported for grapevine and other plant species [72–75].

#### Phytoplasma-infection may cause miRNA-mediated changes in plant morphology and architecture

Plant morphological changes can be attributed to changes in the expression of certain transcription factors, as well as regulatory changes at a post-transcriptional and epigenetic level. The miR156/157 family, which is highly conserved in plants, can target numerous members of the SBP-box genes in *V*. *vinifera*. Evidence has shown that changes in the expression levels of these genes play a role in phase transition and reproductive development [76,77]. Studies on *Arabidopsis* and rice showed that cleavage of squamosa-promoter binding-like (SPL) proteins, due to miR156 overexpression, give rise to plants that are smaller, show delayed flowering and loss of apical dominance, initiate growth of more leaves with shorter plastochrons (in *Arabidopsis*) and causes reduced panicle size (in rice) [78,79]. Likewise, miR156-overexpression in poplar (*Populus* spp.) caused an increase in leaf size and leaf initiation rate, and reduced apical dominance [80]. The modification of leaf morphology due to regulation of SBP-box transcripts by miR156 overexpression was demonstrated in phytoplasma-infected Mexican lime trees, mulberry, and red date [52,81,82]. Expression analysis in this study, however, revealed a significant decrease in abundance of certain vvi-miR156 members in the phytoplasma-infected samples, which cannot be explained at this point.

It has been shown that down-regulation of miR156 results in an increase in SPLs that promote juvenile to adult phase transition and flowering through activation of miR172 and MADS box genes in *Arabidopsis* [83,84]. The *Arabidopsis AtSPL9*, can positively regulate the expression of miR172, demonstrating the presence of a miR156-AtSPL9-miR172 regulatory cascade [85]. It was proposed that the miR156-SPL-miR172 regulatory pathway was activated in mulberry in response to phytoplasma infection [81]. Higher levels of miR172 associated with viral pathogenesis in tomato leaf curl disease and grapevine leafroll disease have also been reported [86,87]. The APETALA2/Ethylene-responsive transcription factor (*AP2/ERF*)-like mRNA was identified as a possible target of vvi-miR172 in our study. The interaction between miR172 and *AP2/ERF*-like targets is well conserved and are known to be involved in transitions between developmental stages, regulating flowering time and specifying floral organ identity [88,89]. Differential expression of vvi-miR156 and vvi-miR172, leading to restricted phase transition, may lead to symptoms associated with GY such as abnormal leaf shape and size, as well as downward curling of leaves and flower abortion. It was suggested that expression changes of miR156 and miR172 may lead to development of green leaf-like structures instead of flowers, also referred to as phyllody, as well as flower sterility in phytoplasma-infected red date and mulberry [81,82].

Levels of vvi-miR159 and vvi-miR319 were also significantly higher in the AY phytoplasma-infected leaves, and *in silico* analysis predicted that they may target a *GAMYB*-like mRNA and a *R2R3-MYB* mRNA. Recent studies identified their association with plant disease during fungal and bacterial infection in *Arabidopsis* and *Populus trichocarpa*, respectively [90,91], and have experimentally validated their targets as being mRNAs encoding MYB transcription factors [92]. MYB genes constitutes a large and widespread gene family in plants (estimated at 279 members in grapevine, of which 108 belong to the R2R3 family), and are involved in a variety of plant-specific functions including primary and secondary metabolism, cell fate and differentiation, developmental processes and responses to biotic and abiotic stresses [93,94]. Consequently, altered expression of miR159 and miR319 may also contribute to deformation of grapevine leaves [82,86].

#### Differential miRNA expression may lead to modulated auxin signalling

Disease symptoms caused by certain pathogens have also been described as a result of interference with plant hormone signalling that lead to the disturbance of plant defence responses. Phytoplasma diseases have been classified as ‘auxonic diseases’ which refers to possible interactions with the auxin balance of the host [95]. Auxin, an important phytohormone, regulates many plant developmental processes, and its influence during pathogen resistance responses has been described [96]. A substantial increase of indole-3-acetic acid (IAA) was observed in phytoplasma-infected Mexican lime trees, possibly indicating susceptibility to the pathogen [52]. Certain proteins, known as virulence effectors, are secreted by pathogens during infection and are known to modulate hormone and signalling pathways by altering gene transcription levels. AY-WB effectors, SAP11 and TENGU, are known to be unloaded from the phloem sieve cells to the target cell nuclei where they interact and destabilise certain transcription factors, resulting in severe changes in leaf morphology and increased susceptibility to phytoplasma insect vectors [97–99]. Microarray analysis of transgenic *Arabidopsis* lines overexpressing TENGU demonstrated regulation of several auxin responsive genes and auxin efflux carrier genes. SAP11 destabilises CINCINNATA (CIN)-TEOSINTE BRANCHED1, CYCLOIDEA, PROLIFERATING CELL FACTOR (TCP) transcription factors 1 and 2, known to be regulated by miR319 in *Arabidopsis*, resulting in the suppression of Jasmonate (JA) production that create favourable conditions for insect vector proliferation [97–99].

A group of miRNAs can promote plant defence responses by coordinate regulation of hormone signalling pathways in response to pathogen attack. Among them, miR160, miR167, miR390 and miR393 contribute to PTI by regulating the expression of genes encoding different auxin response factors (ARFs) and auxin receptors involved in auxin signalling, thereby promoting inhibition of pathogen growth [90]. miR393 expression, induced by bacterial elicitor flg22, was the first shown to be implicated in the repression of auxin receptor genes in *Arabidopsis* [23]. Our results showed that vvi-miR160, which may target *ARF* mRNAs, was significantly up-regulated in the AY phytoplasma-infected leaves. ARF transcription factors are known to regulate auxin-inducible genes by binding to elements in their auxin-responsive promoters to either activate or repress transcription [100]. Other instances where miR160 accumulated during biotic stress response were demonstrated in clubroot-infected *Brassica napus* root [101], powdery mildew infection in wheat [102], and phytoplasma-infected mulberry [81].

#### Phytoplasma-responsive miRNA expression may play a role in nutrient homeostasis

The AY phytoplasma chromosome is extremely reduced and lacks many essential genes related to amino acid and fatty acid biosynthesis, the tricarboxylic acid cycle and oxidative phosphorylation. This suggested that phytoplasmas have evolved as intracellular parasites in nutrient-rich host environments and therefore possess multiple transporter genes in order to assimilate important mineral nutrients for their survival [103]. Several plant miRNAs have been reported for their role in nutrient homeostasis in response to deficiencies of phosphate, nitrogen, sulphur, and copper [104]. A few of these, including vvi-miR395 and vvi-miR399, were differentially expressed in the present study, possibly in response to AY phytoplasma-infection. miR395 is known to target members of the *ATP-sulphurylase* (*ATPS*) gene family and a low-affinity sulphate transporter gene *SULTR2;1*, both crucial for regulating sulphate homeostasis in *Arabidopsis* [105] (S6 File). The induction of miR395 levels leads to sulphate accumulation in the leaves due to increased translocation from the roots [106,107]. In this study miR395 was up-regulated in the AY phytoplasma-infected leaves, and may contribute to favourable conditions for pathogen growth. Alternatively, sulphur starvation can cause physiological imbalances, impaired plant growth, and reduced plasticity against environmental changes and pathogen attack [108]. The role of miR399 in the maintenance of phosphate homeostasis has been well characterised. It is involved in the regulation and allocation of inorganic phosphate (Pi) from the roots to the shoots as well as remobilisation from old to young leaves [109,110]. miR399 positively regulates Pi uptake and translocation by down-regulating *PHO2*, which encodes a ubiquitin-conjugating E2 enzyme, UBC24 [109,111]. *PHO2*, on the other hand, acts as a negative regulator by suppressing these activities when external Pi is ample, thereby preventing phosphate toxicity. Our results revealed significant down-regulation of vvi-miR399 in AY phytoplasma-infected leaves. Interestingly, lower levels of miR399 was also found in phytoplasma-infected material of Mexican lime trees, mulberry, and red date [52,81,82]. An adequate supply of Pi is required for optimal growth and reproduction due to its involvement in essential plant functions, including energy transfer, photosynthesis, enzyme regulation, metabolite transport and nucleic acid synthesis. Therefore, the down-regulation of miR399 may cause suppression of Pi uptake, further contributing to disease symptom development.

### Identification of putative targets for differentially expressed novel miRNAs

In addition to the targets of known miRNA, we also predicted possible targets for the *in silico* predicted novel miRNAs that were significantly differentially expressed in the AY phytoplasma-infected leaves (S6 File). Some of these target mRNAs encode certain transcription factors, such as Scarecrow-like/GRAS-domain protein, TPR-like protein, MADS-box protein, bHLH-like protein, and a NAC-domain protein. We also identified targets that encode proteins involved in hydrolase activity, e.g. ARM repeat superfamily isoform 2-like protein, beta-fructofuranosidase, glucan endo-1,3-beta-glucosidase, and a calcineurin-like metallo-phosphoesterase. Receptor-like kinase (RLK) proteins that are involved in signal transduction, such as a G-type lectin S-receptor-like serine/threonine-protein kinase, a leucine-rich repeat (LRR) receptor EXS-like kinase, a disease resistance At3g14460-like protein, a RLK HSL1-like protein, and a LRR receptor-like serine/threonine At4g08850-like kinase were also identified.

Some signal transduction proteins are surface-located, transmembrane receptor molecules that are activated by external stimuli, such as plant hormones and pathogens. These, in turn, are sequentially transmitted to initiate complex downstream signalling pathways that induce PAMP -and effector-triggered immunity (PTI and ETI) and/or hypersensitive cell death resistance responses. The majority of these innate immune receptors are proteins that contain a nucleotide-binding site (NBS) and leucine-rich repeats (LRR) that are encoded by resistance (*R*) genes [112]. Another versatile function of certain miRNAs is targeting diverse members of NBS-LRRs which are then processed by RNA-dependent RNA polymerase 6 (RDR6) to dsRNA and then cleaved by DCL4 to produce phased, secondary siRNAs (phasiRNAs) [113]. This was demonstrated in resistant *Solanum lycopersicum* (tomato) and *Gossypium raimondii* (cotton) where the miR482-mediated silencing cascade was suppressed in pathogen-infected plants so that certain NBS-LRRs were up-regulated to confer resistance [114,115]. Interestingly, vvi-miRn027, which may target a disease resistance mRNA (*GSVIVG01027229001*), was severely increased in AY phytoplasma-infected leaves in comparison to the other novel miRNAs that might target RLK mRNAs. This miRNA and its putative target may serve as potential candidates in transient expression studies to investigate an underlying defence response to AY phytoplasma.

Furthermore, most of the other novel miRNAs were expressed at a lower abundance than that of conserved miRNAs and are likely to be grapevine-specific miRNAs, which may be classified into non-conserved miRNAs. It can be suspected that they are likely candidates involved in developmental, metabolic and transmembrane transport processes as proposed by the gene ontology results, but it would require additional experimental approaches to address these hypotheses.

## Conclusions

In summary, our study employed different computational tools to provide the first report on the identification of differentially expressed miRNAs in grapevine leaves infected with AY phytoplasma. In addition to known vvi-miRNAs, we detected a large group of putative novel miRNAs by utilizing two different analysis pipelines. Some of the novel miRNAs shared a high degree of homology with other known plant miRNAs, and were therefore classified as newly-identified members of existing miRNA families. Further experimentation concerning the regulation of their target mRNA(s), however, would be required to confirm this.

Differential expression analysis was done via comparative miRNA profiling between sRNA libraries constructed from healthy control plants and plants diagnosed with AY phytoplasma, respectively. Changes in the expression of various miRNAs were clearly observed in the diseased group, possibly modulated in response to biotic stress. The relative expression of certain known and novel miRNAs was determined with real-time RT-qPCR analysis, thereby demonstrating a similar trend in expression regarding the normalised sRNA read data. There is increasing evidence for the involvement of miRNAs in plant-microorganism interaction and how they mediate gene expression related to pathogenesis.

In order to identify potential miRNA targets, we applied a simple complementary-based, *in silico* approach with psRNAtarget. This method relies on perfect or near-perfect complementarity of plant miRNAs with their target(s), known to facilitate gene regulation through mRNA cleavage or translational inhibition. To further validate grapevine-specific miRNAs and the mRNAs they target would require the use of stable and robust degradome sequencing data that would assist in the elucidation of different modes of regulation in a tissue-specific and developmental stage-specific manner. Target mRNAs regulated by translational inhibition, however, would be undetectable in degradome data. Furthermore, high-throughput gene expression profiling techniques such as microarray-hybridisation analysis and RNAseq/transcriptome analysis would allow us to observe expression levels of miRNAs and their anti-correlated target mRNAs.

The miRNA expression patterns observed in AY phytoplasma-infected grapevine leaves, followed by putative miRNA target description and annotation, led us to believe that our results were compatible with evidence of perturbations found in other pathogen-infected plants. Putative miRNA target predictions indicated the involvement of miRNA pathways that may influence plant development and morphology either directly or by auxin imbalance. We also identified targets involved in nutrient homeostasis, as well as a few important novel miRNA targets involved in signal transduction, which may hold the key to activating pathogen-resistance pathways in grapevine. Taken together, our findings suggest some hypothetical associations between miRNAs and certain physiological changes that may be crucial in understanding disease symptom development in AY phytoplasma-infected grapevines. Further investigations of these miRNA-mediated pathways may shed new light on the roles and mechanisms of miRNAs in plant pathogenesis.

## Supporting information

S1 Fig Hairpin structures of differentially expressed novel vvi-miRNAs. (PDF)

S1 File Virus RT-PCR primer list. (XLSX)

S2 File IsomiR summary for each of the mature vvi-miRNAs detected in the healthy and AY phytoplasma-infected libraries. (XLSX)

S3 File Additional differentially expressed known miRNAs. (PDF)

S4 File Putative novel vvi-miRNAs. (XLSX)

S5 File miRNA real-time RT-qPCR primer list. (XLSX)

S6 File psRNATarget AND GO analysis results. (XLSX)
