# Supplementary material for: The use of high-throughput small RNA sequencing reveals differentially expressed microRNAs in response to aster yellows phytoplasma-infection in Vitis vinifera cv. ‘Chardonnay’
Source: PLoS One. 2017 Aug 16;12(8):e0182629. doi: 10.1371/journal.pone.0182629 (PMC5558978; doi:10.1371/journal.pone.0182629)
Supplement: S1 Fig — (PDF) [file pone.0182629.s001.pdf]

Figure S1. Hairpin structures of the differentially expressed novel miRNAs. The sequences highlighted in green and magenta represents the 5' and 3' miRNA sequences, respectively.

>vvi-miRn011.2

```
      A   AAUGA       AAGAUAAAGAUU     GA     ---CAAAAGAU    G -        C   UC             C  C U    CAU    AU
UCUAUA AUA     CCUCUGC            CCAGU  UCCUA           UGCA A GAAAGGUU UCU  GCAGGAGAGAUGA GC G CGCC   GUCC  U
|||||| |||     |||||||            |||||  |||||           |||| | |||||||| |||  ||||||||||||| || | ||||   ||||
AGAUGU UAU     GGAGACG            GGUCG  GGGGU           AUGU U CUUUCCAA GGA  CGUCCUCUUUACU CG C GCGG   CGGG  A
      A   ----A       ------------     -A     CAAAACUGAAU    G A        A   UA             A  A C    -AU    AG
```

>vvi-miRn025a (vvi-miR171_new)

```
      U  CU   GC         C       C      A     -       ACACUUCCAA
AUGAAA GG  GCU  GAUAUUGGC CGGUUCA UCAGAU CAGGA UACACCU          A
|||||| ||  |||  ||||||||| ||||||| |||||| ||||| |||||||
UACUUU CC  UGA  CUAUAACCG GCCGAGU AGUUUA GUUUU AUGUGGA          A
      C  -U   GU         U       U      -     C       AAAAAAAAAA
```

>vvi-miRn007.1

```
   A  G G    --C G  G  -    --C       AU    U   ----------C    CGGA     UAA  G     A           A            G        AU       C        GGAUCAG  UAC
UCG UC U UUUC   C UU CU CCAG   CAUUUUG  AGUU GGU           CAUC    AGAUU   GG AAAGU UGUUCUCAGCU CUAAUAUCAAGA AAUGAGAA  UGAGAAC CAACUUUG       CA   U
||| || | ||||   | || || ||||   |||||||  |||| |||           ||||    |||||   || ||||| ||||||||||| |||||||||||| ||||||||  ||||||| ||||||||       ||   U
AGU AG A AAAG   G AA GA GGUU   GUGGGAC  UCGA CCG           GUAG    UUUAG   CC UUUCA ACAAGAGUCGA GAUUAUAGUUUU UUACUCUU  GCUCUUG GUUGAAAC       GU   C
   G  A G    AAA G  G  A    GUU       AC    -   GUGUUGAAAAU    UAAA     UAC  G     C           C            A        --       A        GAUAAAA  UCC
```

>vvi-miRn117

```
     C     A   C         C      GA G UC       U      A U   C A AC                                      C  AGAAAAGUUCAUUGCAUAUACAG
GAAUC ACUUU AUA CAUUUGUAA GGUUUG  C U  UUGUGUA GUAUUG C GCU U G  UCAAUGGACCCUCAUGACUUUAAAAUGCAUCUACAUAA UA                       U
||||| ||||| ||| ||||||||| ||||||  | |  ||||||| |||||| | ||| | |  |||||||||||||||||||||||||||||||||||||| ||
CUUAG UGGGA UAU GUAAACGUU CUGAAC  G G  GGCACAU UAUAAU G UGA A C  AGUUACCUGGGAGUGCUGAAAUUUUGUGUAGAUGUAUU AU                       U
     A     C   A         A      GA G UA       C      A U   A C CA                                      A  CCCAUUUCCCCUUUUCAAGGAUU
```

>vvi-miRn022b (vvi-miR1446_new)

```
       ----               C   C        U        A     G   GAACA  U
AGGGAGA    CUGGCAUUUUCUGAA UCU UCCCUCAU GGCUUUCU AGCUU UCU     UC A
|||||||    ||||||||||||||| ||| |||||||| |||||||| ||||| |||     ||
UCUCUCU    GGUUGUAAAAGACUU AGA AGGGAGUG CCGAAGGA UCGAA AGG     AG G
       UUUA               U   -        -        G     G   -GAAA  A
```

>vvi-miRn008.1 (vvi-miR169_new)

```
    -   -G          -   A  A      -  U   C    U  GU   -        A     U     CUCCUCCCGAGUGGUUCGUGGGGAU
GAAU UUG  GAGGAAGAGA GGU UA CAUGAA GA AAG GCCU GU  GGU AGCCAAGG UGACU GCCUG                          A
|||| |||  |||||||||| ||| || |||||| || ||| |||| ||  ||| |||||||| ||||| |||||
CUUA AAC  CUUCUUCUCU CCG AU GUACUU CU UUC CGGA CA  UCA UCGGUUCC ACUGA CGGAC                          G
    U   CA          U   G  C      U  C   A    -  GU   A        -     -     UCCUGGUGUGUCCCCAGAUAUUAGA
```

>vvi-miRn040

```
      ---------AGG U   -     UCCAUACAU                       A      C     C    AA U       A     ----   --A    C   U    CAUC        UA    GA    A
GCUUAC            G GCC AAAUU         UUCGUAUAAAUAUUGUCUGCUUU GGUCCA UAGCC UCAC  C UUAAAAU UGUCU    ACA   AUAC AGA GAGU    CCUUAUAA  GUGU  AAGA A
||||||            | ||| |||||         ||||||||||||||||||||||| |||||| ||||| ||||  | ||||||| |||||    |||   |||| ||| ||||    ||||||||  ||||  ||||
CGAAUG            C CGG UUUAA         GAGCAUAUUUAUAACAGAUGAAA UUGGGU AUCGG AGUG  G AAUUUUG GUAGA    UGU   UAUG UCU CUCA    GGAAUAUU  UACA  UUCU U
      AAACUUGGGAAC -   C     --CAAUAAU                       C      A     A    CG U       C     UUGA   GCC    A   U    -ACA        --    -G    C
```

>vvi-miRn051

```
        -U          C A                          ACU  -      A A      C C          A  U   A U     U              G           C        A        GC C                 G   GAAGUUCUUUAUGGAAUUGCCUCAACC
ACCUACUA  UGUCAUUGUU A AAUUAUGUCGGAGGUGGCUUCUCAAA   UG AAAUUC A GAGGUU U UUUGUGGUCA GA UCA U AAGGU GGAGAGACCACCUA UCCUGUUAAGA CAAGUUUA GGGCCUAA  U GAGAGGGGCUUAGGUUU GGG                           A
||||||||  |||||||||| | ||||||||||||||||||||||||||   || |||||| | |||||| | |||||||||| || ||| | ||||| |||||||||||||| ||||||||||| |||||||| ||||||||  | ||||||||||||||||| |||
UGGAUGAU  ACAGUAACAA U UUAAUGUAGUCUCCACCGAAGAGUUU   AC UUUAAG U CUCUAA A AAACACCAGU CU AGU A UUCCG CCUUUCUGGUGGGU AGGACAAUUCU GUUCAAAU UCUGGAUU  G UUCUCCCCGAAUCCAAA CCC                           U
        GU          A C                          AAC  U      A C      - A          C  U   C C     U              G           A        A        AA A                 A   UCAAUCCCUUAUUUUGGAAUAACCUUU
```

>vvi-miRn025b (vvi-miR171_new)

```
   CC    CG      U    U  U     U         U       C     - A      A     GAAAAAACAAAC
CAA  GUGA  AGGAGU GAAA AG UACUG GAUGUUGGC CGGUUCA UCAGA C AAAAGA AAAAA            A
|||  ||||  |||||| |||| || ||||| ||||||||| ||||||| ||||| | |||||| |||||            C
GUU  UACU  UCUUUA CUUU UC GUGAC CUAUAACCG GCCGAGU AGUUU G UUUUCU UUUUU            A
   --    CA      -    -  C     U         U       U     C A      C     ACGUACGUAUAU
```

>vvi-miRn131 (vvi-miR399_new)

```
          CA  A U      U A    A        U   -A    -  CU   -G    UU
UGCAUGGAUG  UU C GGGCAA U CUCC UUGGCAGU GGC  ACUC GG  GAC  GUGA  A
||||||||||  || | |||||| | |||| |||||||| |||  |||| ||  |||  ||||
ACGUGUCUAC  AA G CCCGUU A GAGG AACCGUCA UCG  UGAG CC  UUG  CACU  U
          UC  C C      U -    A        C   AA    A  --   AA    UC
```

>vvi-miRn003

```
         G      CA  C         U          UUUGUC
AGGAUCCAU CCAUUU  UUA ACAGAGAGA GACGGUGGAGA      U
||||||||| ||||||  ||| ||||||||| |||||||||||
UUCUAGGUG GGUAGA  AAU UGUCUCUUU CUGCCAUCUCU      A
         A      CG  A         -          CGUUAU
```

>vvi-miRn010.2 (vvi-miR529_new)

```
     C     -  CCU  A  A          A        AUAC       CC
GUGUC AUCAA GA   GC GG GAAGAGAGAG GUACAGCU    UUGCUCC  G
||||| ||||| ||   || || |||||||||| ||||||||    |||||||  U
UAUAG UAGUU CU   UG CC CUUCUCUCUC CAUGUCGA    AACGAGG  U
     -     U  AUU  C  C          C        AGGU       UA
```

>vvi-miRn022a (vvi-miR1446_new)

```
-CU             CU     A    C     A      A   GAAUGU
   UUAUCUGAACUCU  CCCUC UGGC UUUCU AGCUUU UCC      C
   |||||||||||||  ||||| |||| ||||| |||||| |||      U
   AAUAGACUUGAGG  GGGAG ACCG AAGGG UCGAAG AGG      A
UGU             -U     A    -     G      -   AAAGAA
```

>vvi-miRn003

```
--UUUCA   C         U           UUUGUC
       UUA ACAGAGAGA GACGGUGGAGA      U
       ||| ||||||||| |||||||||||
       AAU UGUCUCUUU CUGCCAUCUCU      A
GUAGACG   A         -           CGUUAU
```

>vvi-miRn137

```
----C    -  A     -A    AACA    UCUUAAUAACUU       UCCAGCAA
     AACA AC AUCUA  AUGA    UAGA            AUACUUC        C
     |||| || |||||  ||||    ||||            |||||||        A
     UUGU UG UAGAU  UACU    AUCU            UAUGAAG        A
AAUAA    U  A     GG    ----    ---------UUA       UAAAUUAA
```

>vvi-miRn152

```
--                        U
  UAGAUAGACUGGUAGAAAGAUUUA A
  ||||||||||||||||||||||||
  AUCUAUCUGACCAUCUUUCUAAAU U
CU                        A
```

>vvi-miRn133

```
--UUG    AA  AG       --   G   --     GAU
     AUCG  CA  UCAGCAA  UGA CGG  AAUAG   A
     ||||  ||  |||||||  ||| |||  |||||
     UGGC  GU  AGUUGUU  ACU GCC  UUAUU   U
UCUGA    CC  -G       CC   -   AU     GUU
```

>vvi-miRn129

```
           U                 U        U             CAC
AUGUGAGCAAG GAGAGACAUUUUGGAAC AGAGUGCU GCAUUUAUUUAAA   C
||||||||||| ||||||||||||||||| |||||||| |||||||||||||   A
UACGUUCGUUC CUCUCUGUAAAACUUUG UCUCACGA CGUAAAUAAAUUU   A
           U                 U        U             UAC
```

>vvi-miRn070

```
      C  G    C        C                  A     CAACAAC     A
UGAAAU AA UUGG CUACCAAU UAGUUUUUAGGGUUUGUG GGUUU       GUUAC U
|||||| || |||| |||||||| |||||||||||||||||| |||||       ||||| G
ACUUUA UU AACC GAUGGUUA AUCAGGAAUCCCAAACAC CUAAA       CGAUG G
      A  A    A        A                  C     ----ACU     A
```

>vvi-miRn115

```
                        A             A
CUAUGCCAAGAAGCACAUUCCUCC AUCUAACUUCCCU G
|||||||||||||||||||||||| ||||||||||||| G
GAUACGGUUCUUCGUGUAAGGAGG UAGAUUGAAGGGA A
                        A             A
```

>vvi-miRn089

```
             C   -           --- -             C       -GC           C   A     A            U           G    C          AAGUGUAUGAUU
..AAUUCAAAUGC UAU CCUAAAGGAUG   C AUCUCUUGCAAAC UAAACCU   CCGGUCCGCAC AAC CACGU UUAUUAAAGUGU ACACAUGUAGU CCAU AUAUGAAGAA            U
  ||||||||||| ||| |||||||||||   | ||||||||||||| |||||||   ||||||||||| ||| ||||| |||||||||||| ||||||||||| |||| ||||||||||            U
..UUAAGUUUGCG AUA GGGUUUCCUAC   G UAGGGGAUGUUUG AUUUGGA   GGCCAGGUGUG UUG GUGUA AGUAGUUUCACA UGUGUACAUCA GGUA UAUAUUUCUU            U
             A   C           AUA A             A       GAG           A   G     C            U           G    U          GACUAACUAAUU
```

>vvi-miRn027

```
                                        C        C                                                AGUAC
..UAUUUUCACACGAAACUAAAUAAAUGAGUAAUAGACAA GAAACAAC AGAUCACAAGAAAACAAAGAAAUCAACAUAAUGAAAAAUAAUUUUGCU     A
  |||||||||||||||||||||||||||||||||||||| |||||||| ||||||||||||||||||||||||||||||||||||||||||||||||     G
..AUAAAAGUGUGCUUUGGUUUAUUUACUCAUUAUCUGUU CUUUGUUG UCUAGUGUUCUUUUGUUUUUUUAGUUGUAUUACUUUUUAUUAAAACGA     A
                                        A        U                                                CAUAU
```

>vvi-miRn139

```
-A  A  -  -    --U    A            -----       -  GU
  AG GC CU CGAC   UCGG CCUUCAUGGGGG     GGCUGAG CU  U
  || || || ||||   |||| ||||||||||||     ||||||| ||  G
  UC CG GA GUUG   AGUC GGGGGUGUUUCC     CCGACUC GA  G
AC  -  A  A    UCC    -            AGGUU       C  GG
```

>vvi-miRn150

```
-CC      -----   AU         -AG    ---        --GG  -  GGUU
   GCUUUU     UUC  GGUCUGAUU   GCUG   AAAUUUGG    GA GA    U
   ||||||     |||  |||||||||   ||||   ||||||||    || ||
   UGAAAG     GAG  UUAGGCUAG   UGGC   UUUAAAUU    UU CU    A
UAC      GUUUU   AU         AAG    AAG        AUUA  A  GAAU
```

>vvi-miRn147

```
--TUU   G  G   C   U             U  G  --A     UC
     AAU GU AAC AAA AACUCUGGUUAUG UG UC   AUAUG  A
     ||| || ||| ||| ||||||||||||| || ||   |||||  C
     UUA CA UUG UUU UUGAGACCAAUAC AC AG   UAUAC  U
AAGAC   G  G   A   C             U  G  AGG     UA
```
