# Supplementary material for: The use of high-throughput small RNA sequencing reveals differentially expressed microRNAs in response to aster yellows phytoplasma-infection in Vitis vinifera cv. ‘Chardonnay’
Source: PLoS One. 2017 Aug 16;12(8):e0182629. doi: 10.1371/journal.pone.0182629 (PMC5558978; doi:10.1371/journal.pone.0182629)

vvi-miRNA	Sequence (5'-3')	Length (nt)	Avg of normalised read counts†		DESeq results (H vs AY)		
			H	AY	log2FC	*p*-value	Adj. *p*-value (*q*-value)
vvi-miR319b,c,f	UUGGACUGAAGGGAGCUCCCU	21	527.50	1811.90	1.31	0.027	0.150
vvi-miR319e¶	UUUGGACUGAAGGGAGCUCCU	21	6.07	25.24	1.49	0.008	0.108
vvi-miR3627-5p	UUGUCGCAGGAGAGACGGCACU	22	1.89	5.05	1.22	0.009	0.060
vvi-miR3629ab-3p,c-5p	GGCUGCUGAGAAAAUGUAGGA	21	25.95	8.95	-1.15	0.031	0.150
vvi-miR395a,b,c,d,e,f,g,h,i,j,k,l,m	CUGAAGUGUUUGGGGGAACUC	21	1642.99	6002.63	1.23	0.049	0.220
vvi-miR399a,h	UGCCAAAGGAGAAUUGCCCUG	21	10.08	2.97	-1.34	0.016	0.100
vvi-miR399e¶	UGCCAAAGGAGAUUUGCCCGG	21	463.24	103.94	-1.55	0.009	0.060
vvi-miR479¶	UGUGGUAUUGGUUCGGCUCAUC	22	71.26	24.82	-1.16	0.030	0.150
vvi-miR3638-5p	UGUGCCUUUUCGCGCUUGUUGCUA	24	1.57	0.25	-0.88	0.049	0.115

H: Healthy sample group

AY: AY phytoplasma-infected sample group

¶Validated using real-time RT-qPCR

†Average of reads per million mapped reads (RPM) between three biological replicates

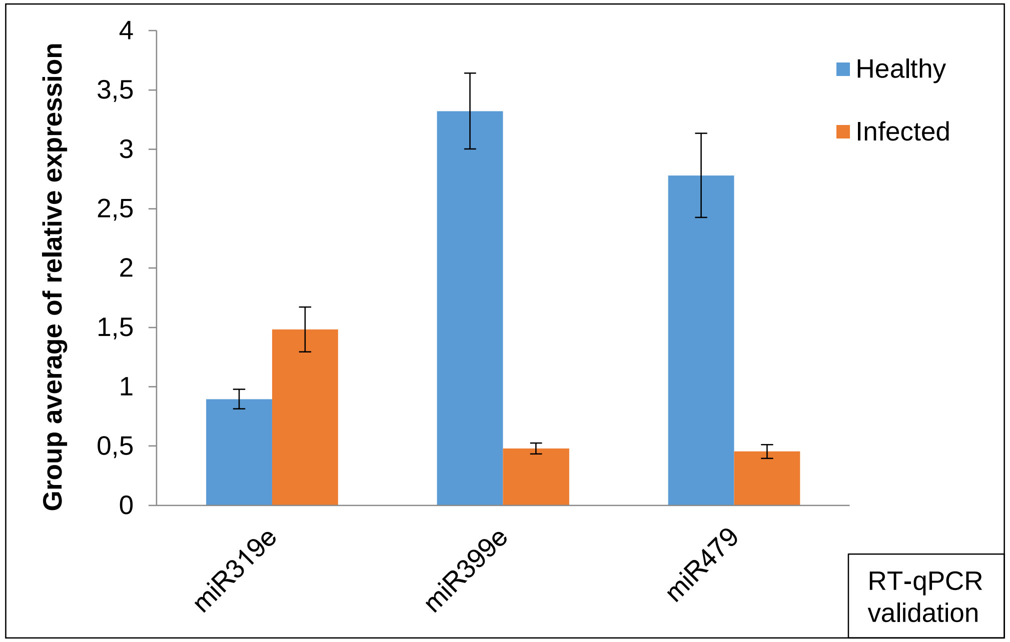

Supplement: S3 File — (PDF) [file pone.0182629.s004.pdf]
